# Native and non-native lexical processing in Mandarin: pinyin and tone representation

**DOI:** 10.3389/fpsyg.2026.1856709

**Published:** 2026-07-16

**Authors:** Marcus Taft

**Affiliations:** School of Psychology, University of New South Wales, Sydney, NSW, Australia

**Keywords:** Chinese as a foreign language, Chinese lexical processing, Chinese reading, Chinese tones, pinyin

## Abstract

The nature of the Chinese mental lexicon is described in this theoretical paper, with a particular focus on the way in which Mandarin is represented and processed, especially with regard to tonal information. Because an alphabetic script (i.e., pinyin) is adopted in China at the earliest stages of reading acquisition, an account is given of the way in which alphabetic orthography is processed, using English as the example. Following from this, a framework for thinking about the processes involved in learning to read Chinese is presented and the impact of pinyin is considered. In particular, it is argued that knowledge of pinyin has an influence on the phonological component of the lexical processing system, especially in terms of separating the tone from the rest of the syllable. Past studies of Mandarin and Cantonese are used to provide evidence for the argument that pinyin modulates the phonological representation, with the added claim that the phonological system exists in order to generate a pronunciation. Spoken words are recognized through a separate system that is sensitive to the surface form, and it is here that there is a critical difference between native and non-native Mandarin speakers, especially those with a non-tonal first language. The inability to identify lexical tone in the speech signal, along with the representation of tone in pinyin as a diacritic separate from the letters, means that the non-native speaker has difficulty remembering what the correct tone is for any word they are trying to learn. The depiction of the lexical processing system that is presented in this paper is meant to provide a concrete framework for helping to understand the way in which both native and non-native speakers process Chinese words, especially when reading.

## Introduction

Chinese is a morphosyllabic language whereby most morphemes are represented by a single syllable and the majority of words are composed of more than one morpheme. Every morpheme is composed of a syllable plus a lexical tone. Mandarin, the standard version of Chinese spoken in China, has four different tones, each of which provides a different meaning to the same syllable. For example, the syllable /maʊ/ spoken with a level tone (referred to as Tone 1) means “cat,” but when spoken with a rising tone (Tone 2) it means “hair” (among other things) and with a falling tone (Tone 4) it means “cap” (among other things).

When it comes to the orthographic representation of Chinese, each morpheme corresponds to a single character which is often composed of subunits referred to as “semantic” and “phonetic” radicals. Unlike in an alphabetic script where the orthographic subunits are letters, the pronunciation of Chinese characters is not systematically related to their subunits, with only a small percentage of characters being pronounced identically with their phonetic radical. This means that when Chinese children learn to read, they need to memorize the association between a character and its pronunciation, unlike in an alphabetic script where the pronunciation of many words can be correctly generated via the conversion of their letters into sounds (e.g., the pronunciation /kæt/ can be generated from *CAT* via the conversion of *C* to /k/, *A* to /æ/, and *T* to /t/). One major advantage of alphabetic script, then, is that once the rules of letter-sound conversion are learnt, the child can self-generate the pronunciation of many words that they know by sound but not by sight, and can even learn the correct orthographic representation from doing this (see, e.g., Share, 1995).

Given its potential benefits, China adopted an alphabetic script in 1958 in an attempt to facilitate the teaching of Chinese characters. This script was named Hanyu Pinyin, or “pinyin” for short, representing the sounds of Mandarin in the form of Roman letters (e.g., *māo* is pinyin for /māʊ/, “cat”). Note that the lexical tone is represented by a diacritic mark above the vowel, though it is also sometimes represented by the number of the tone (e.g., *mao1*). Yuan et al. (2022) outlines the generally accepted rationale for the use of pinyin in terms of it enabling children to access the meaning of visually unfamiliar characters for which they already know the pronunciation. That is, knowledge of pinyin provides a link between speech and writing in Chinese, which is why children in China are taught pinyin at the earliest stages of literacy acquisition. Indeed, studies have demonstrated that knowledge of pinyin does facilitate the reading of characters (e.g., Li et al., 2016; Lin et al., 2010; Wang et al., 2014).

In this theoretical paper, we explore the general nature of the lexical system used by Mandarin speakers when they read, with a particular interest in the involvement of pinyin and the way information about tones is captured. A contrast will also be drawn between the way tones are represented in the lexicon of native Mandarin speakers and in that of non-native learners. By drawing upon a defined model of the pathways involved in lexical processing, the paper is able to make concrete suggestions about the nature of lexical processing and, although empirical evidence is presented, many of the arguments remain speculative. The intended aim of the paper is to provide a springboard for further research by making strong claims that have some empirical support but are potentially controversial.

## Reading acquisition in an alphabetic script

Before they learn to read, children develop the ability to understand speech. That is, they are able to access their semantic system via the sounds of words, which means that the words they know are represented in a phonetically based lexicon corresponding to the pronunciations they hear, even if they are not aware of the individual sounds that make up those words (e.g., Castles and Coltheart, 2004). While this spoken word input system represents a direct auditory interpretation of the acoustic signal, it must allow for sufficient generalization in order that the same word be understood when spoken by different speakers (e.g., Johnson and Sjerps, 2021).

**Figure 1:**
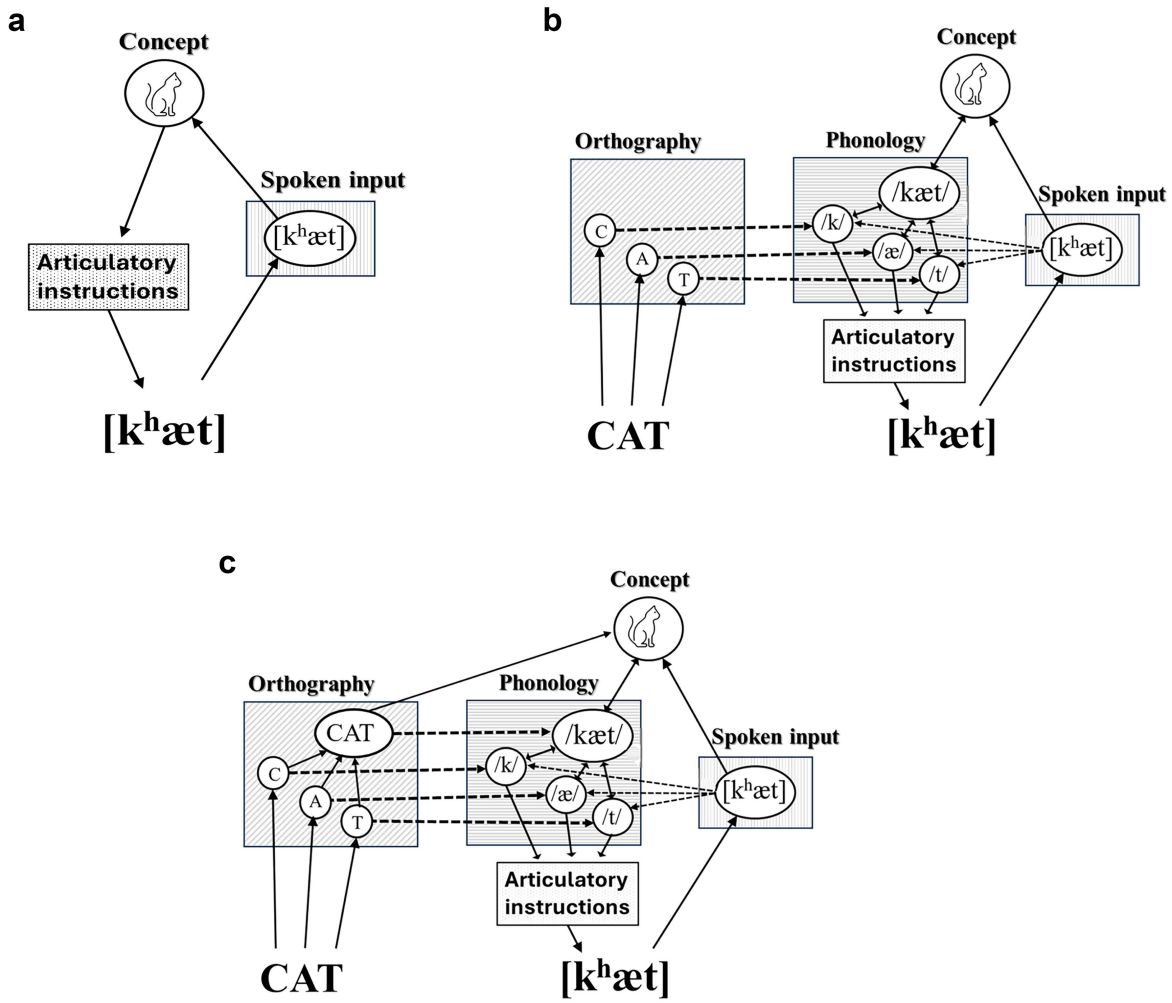
**(a)** Recognizing and producing the English spoken word /k^h^æt/ for pre-literate children. Going from spoken word to concept and vice versa. **(b)** Development of the phonological system at the early stage of literacy acquisition (following Taft, 1991). **(c)** Development of the orthographic system at a somewhat later stage of literacy acquisition where a whole-word orthographic unit develops in association with the whole-word phonological representation.

In addition, children learn to speak, which implies that they can go from the stored meaning of the word to its pronunciation. The simplest way to conceptualize this is for mentally-stored information representing the concept to be directly linked to a set of instructions to the articulators (i.e., how to move the tongue, lips, vocal chords, etc) in order to generate the pronunciation associated with that concept. Figure 1a depicts this pre-literate stage of the system.

When it comes to the stage where the child acquires literacy, the written word needs to be decoded. In an alphabetically scripted language, this initially entails a translation of the individual letters of the word into phonemes (e.g., *C* ➔ /k/, *A* ➔ /æ/, *T* ➔/t/) which are combined to generate an articulatory output. At the earliest stages of reading, this spoken output can be fed directly back into the speech recognition system, which provides access to conceptual information, meaning that the child can only read by speaking the letter-strings aloud (e.g., Gough and Tunmer, 1986). However, if a phonemic representation for the whole word is developed, this provides the opportunity to mediate between the meaning of the word (i.e., the concept) and its articulatory output. As such, the pathway for reading goes from print to sound which is then used to gain access to meaning (e.g., Castles et al., 2018).

It has been proposed that letters (or, more precisely, “graphemes” which includes combinations of letters such as *TH*) generate phonemes through a discrete set of grapheme-phoneme conversion rules (e.g., Coltheart et al., 2001). However, it is also possible to conceptualize this sub-lexical conversion as taking the form of associated links rather than a set of rules. Such a system is depicted in Figure 1b based on the hierarchical model proposed by Taft (1991, 2006a) which provides greater flexibility than rule-based conversion. Here, the printed word *CAT* leads to the activation of orthographic units that are set up for the individual graphemes, and these act as labels for the individual phonemes. As such, the grapheme units *C*, *A*, and *T* activate the phoneme units /k/, /æ/, and /t/, which are then combined and either spoken aloud (and fed to the meaning level through the speech recognition system) or used to activate a phonemic representation for the whole word /kæt/, which provides access to meaning.

Note that the term “phonology” is used to identify the system that contains the phonemic representations, which are abstractions from the surface pronunciation (see, e.g., Pierrehumbert, 1990). That is, while the spoken input system captures the phonetic specifications found in the speech signal (such as aspiration, vowel length, nasalization, etc), the phonological system strips away these details by grouping together variations in the speech signal that do not change the meaning of the utterance. For example, aspirated and non-aspirated versions of the voiceless velar plosive (i.e., [k^h^] and [k^o^] respectively) are categorized as the same phoneme /k/ in English because when one is substituted for the other it does not change the meaning of the word. In this way, the representations in the phonological system consist of psychological entities that correspond to the sounds that make up a word.

An elaboration on the nature of the spoken input system is beyond the scope of the present paper, and is only depicted as a whole-word representation of the phonetic form of the utterance for the sake of simplicity (see Kapnoula et al., 2024, for an overview of the lexical processing of spoken words and Pardo et al., 2021, for an overview of speech perception more generally). The essential point is that the system interprets the speech signal by converting acoustic features into a phonetic realization and provides a link to the more abstract phonological form. These links are depicted in Figure 1b as being below the whole-word level, which captures the fact that spoken nonsense words can be repeated out loud despite them not having a lexical representation.

For all English words to be read, however, the system depicted in Figure 1b is insufficient because only words that follow the most common grapheme-phoneme mappings will have access to the semantic level via a phonological representation. For example, the irregular word *SAID* will be translated into the inappropriate phonemic form /seɪd/ rather than /sɛd/ because the grapheme *AI* is most commonly pronounced /eɪ/ and, therefore, the correct phonological representation /sɛd/ cannot be accessed. For this reason, there needs to be another way of reaching the phonological representation over and above the sublexical (i.e., grapheme-phoneme) level (e.g., Coltheart et al., 2001). For such a “lexical” route to function, an orthographically based lexicon must be developed with representations of the written version of a word that is linked directly to its corresponding representation in the phonological lexicon. This is seen in Figure 1c, where the grapheme units (e.g., *C*, *A*, and *T*) feed into a whole-word orthographic representation (i.e., *CAT*) which, in turn, is linked directly to the whole-word representation in the phonological system. In the final stage of reading development, the orthographic representation is able to directly activate the concept rather than being mediated through the phonological system (see Taft and van Graan, 1998), with both available lexical pathways being depicted in Figure 1c.

## Reading acquisition in Chinese

As can be seen in Figure 1c, then, both sublexical and lexical input is available for reading an alphabetic script such as English. When it comes to reading Chinese characters, however, the sublexical route is not available because characters are not composed of individual letters. As a result, only a lexical pathway can be used. That is, an association must be developed between the orthographic unit representing a character (e.g., 猫) and the meaning of that character (“cat”), and this can be potentially achieved through the involvement of a phonological unit (/māʊ/) representing the word (or, more correctly, representing the morpheme, given that the majority of Chinese words are composed of more than one morpheme/syllable).

The development of the Mandarin phonological representation is likely facilitated by the learning of pinyin. In primary-school textbooks for teaching literacy to native speakers in China, the character representing a known word is presented together with its pinyin transliteration. The child can then use the pinyin to generate the pronunciation of the word which is already associated with a meaning. The corresponding character presented with the pinyin can then be associated with that meaning and its pronunciation, even without the aid of a teacher. If there were no mediating pinyin representation, a teacher would have to individually teach every character by vocalizing the morpheme with which that character is to be associated.

**Figure 2:**
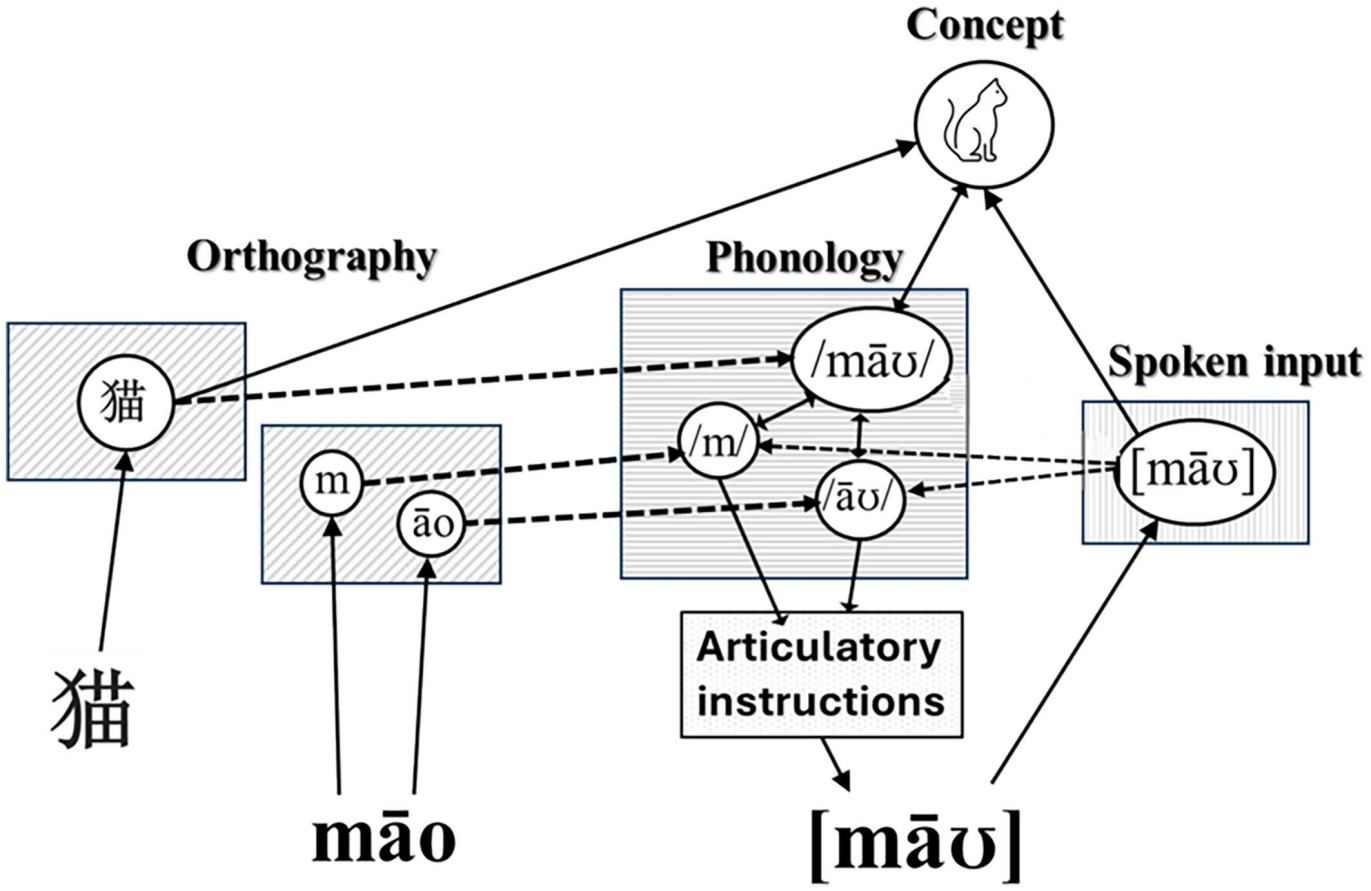
The Mandarin lexical system after pinyin has been used as a mediating tool for learning characters.

With pinyin being employed in this way, it is necessary for the child to develop a phonological system, as with any alphabetic script. This is illustrated in Figure 2 using the example of *māo*, which is the equivalent of the English model seen in Figure 1c but with two separate orthographic systems, one for pinyin and one for characters. Pinyin always allows the pronunciation of the word to be pronounced correctly without any irregular exceptions, which means that the sublexical pathway could potentially be used to generate sound from print in every case. It is nevertheless possible that an orthographic representation for the whole pinyin morpheme is developed as well, hence allowing both the sublexical and lexical routes to be used. However, since the existence of a whole-morpheme pinyin representation is theoretically inconsequential here, it is not depicted in Figure 2. As with any alphabetically scripted language, then, knowledge of pinyin allows the child to access the spoken representation of the words they know, hence allowing the meaning associated with that pronunciation to be accessed. An orthographic unit representing the character presented with the pinyin can then be set up and linked with the relevant concept.^1^

This character unit can potentially be linked directly to the phonological representation as well (as is depicted in Figure 2). However, there exists empirical evidence to suggest that a character unit is actually only linked to its phonological representation via the concept level. In particular, Taft and Zhu (1995) examined a rare type of character that is not itself a morpheme, but rather, one component of a bisyllabic morpheme (which is referred to as a “binding word”). For example, 蚯蚓 (“earthworm”) is a binding word because the two characters are only ever used in combination with each other. Neither character has any meaning on its own. What Taft and Zhu (1995) demonstrated was that, when presented individually, the first character (e.g., 蚯) was associated with shorter naming latencies than the second character (e.g., 蚓) but the two were equally well recognized as characters. The latter result indicates that each character has an equally accessible representation in the orthographic system. In contrast, the naming result suggests that the pronunciation of each character needs to be accessed via a representation corresponding to the whole binding word whereby the first syllable takes precedence in generating the pronunciation. If there were a direct link between the representation of an individual character and its pronunciation, there would be no difference between the naming latencies of the first and second character of such binding words. Instead, it was argued that naming is mediated through the concept level where the whole word is represented.^2^

If this conceptualization is correct, refinements need to be made to Figure 2. In particular, the dotted line connecting the orthographic unit 猫 and the phonological unit /māʊ/ should not be there, and the arrowed line connecting the concept and the phonological unit /māʊ/ should be unidirectional, going only from the former to the latter. In other words, the phonological system exists primarily for the purpose of generating a pronunciation, which means that all access from phonology to the semantic level must be mediated through the spoken input system, even if this is achieved subvocally or covertly.^3^ By this account, then, the phonological system exists to generate output, even if that output can then be used as input into the spoken word recognition system via articulation.

Note that there is an important theoretical issue arising from the idea that phonology does not send direct activation to the conceptual level. According to such an account, conceptual information can only be accessed directly from the phonetic information processed in the spoken input system. However, speech recognition models typically assume that spoken words are recognized via a conversion of the phonetic/acoustic input into a phonological form (see, e.g., Xie et al., 2023) and the lack of a pathway from the phonological representation to the concept appears to preclude this. What can be suggested, though, is that the phonological information activated directly from the spoken input system (see Figure 2) is covertly articulated and this is then fed back into the spoken input system. As such, the activation of phonology can be seen as refining the information that has been input into the spoken word recognition system, with the analysis of the phonetic input being usefully modified by the phonemic interpretation of the signal, hence facilitating access to the correct meaning. Such an idea appears to be consistent with models of speech recognition that center on the integration of auditory and motor processes (e.g., Arjmandi and Behroozmand, 2024) because spoken word identification is being influenced by articulatory output via the phonological system.

## Representation of tones

For the sake of convenience, Figure 2 illustrates the tone accompanying the syllable as being tied directly to the vowel. However, there is ample evidence from a variety of tasks to suggest that tones are actually represented separately from the syllable within the phonological system (see Alderete et al., 2019; O'Séaghdha et al., 2010 for overviews). For example, Chen et al. (2002) used an implicit priming task to show that there was a benefit in generating the pronunciation of a Mandarin syllable when primed by a spoken syllable with the same segments but a different tone, even if this was weaker than when the tone was the same.

Strong support for the idea that a syllable and its tone are separately represented in the phonological system was reported in a study by Taft and Chen (1992) which required the generation of phonology from orthography. In one of their experiments, two characters were consecutively presented to native Mandarin speakers, and the task was to decide whether the second character was pronounced identically to the first (e.g., 保 and 宝 which are pronounced identically, namely, *bǎo* in pinyin). The interesting result was observed with the non-homophones where it proved extremely difficult to say that two characters were not pronounced identically when they differed in their tone (e.g., 区 and 去, which are *qū* and *qù* respectively) compared to when they differed only in their vowel (e.g., 氣 and 去, which are *qì* and *qù* respectively) This was true regardless of whether the task was performed silently (an error rate of 27.8% for the tone-different condition versus 5.2% for the vowel-different condition, and an RT difference for correct responses of 168 ms between these two conditions) or whether the characters were spoken aloud (error rates of 43.1% versus 9.3%, and an RT difference of 90 ms). So, it is apparent that a difference in tone is not as identifiable as a difference in the vowel, even when participants listen to themselves pronouncing the two characters.

A further experiment by Taft and Chen (1992) examined homophone judgments to singly presented characters when other characters existed with the same segments but a different tone (e.g., 肉 is the only character pronounced *ròu*, but there are other characters pronounced *róu*, e.g., 柔) compared to when no other characters shared the same segments (e.g., 丢 is the only character pronounced *diū*, and there are no characters pronounced *diú*, *diǔ*, or *diù*). Deciding that the *ròu*-type of character was a non-homophone proved to be harder than deciding that the *diū*-type of character was a non-homophone and this was true whether the character was pronounced out loud or not. That is, Mandarin speakers considered a character to be a homophone of another character even when their tones differed.

**Figure 3:**
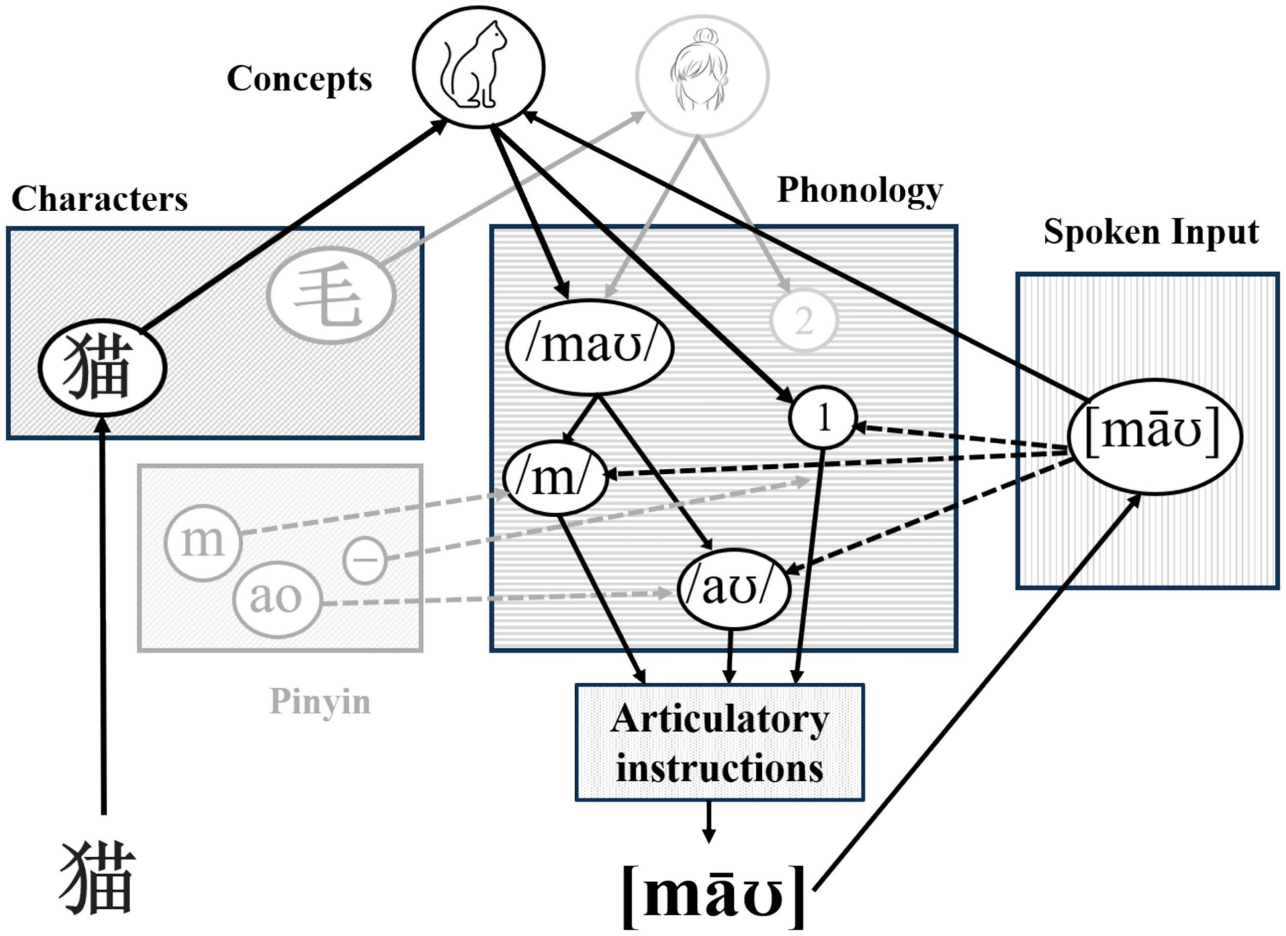
The Mandarin lexical system with tones separated from the segmental information. The morphemes 猫 (“cat”) and 毛 (“hair”) are depicted, where they share the same segments but differ in their tone (*māo* and *máo*, respectively, in pinyin). Phonology is seen as an output system, receiving activation from the conceptual system and not vice versa.

It can therefore be concluded that tones are often ignored when making judgments about the pronunciation of characters which, in turn, implies that phonological representations keep the tone separate from the rest of the syllable. Figure 3 illustrates such a situation and helps explain why the difficulty in determining the lack of homophony between tone-different characters is maintained even when the speaker listens to their self-generated pronunciations.

The examples of 猫 (“cat”) and 毛 (“hair”) are presented in the figure to illustrate the situation where two morphemes share their segments but differ in their tone (*māo* and *máo*, respectively, in pinyin). Mediated through its associated concept, a character representation activates the word level in the phonological system which has separate units for the syllable /maʊ/ and its tone (labeled arbitrarily by the number of the tone^4^). Following from the Taft and Chen (1992) study, it would be hard to say that 猫 and 毛 are not pronounced identically and, according to the proposed account, this would be because they both activate the same syllable unit in the phonological system (i.e., /maʊ/) as a result of the tonal information being represented separately. In order to speak the characters out loud, the phonological system generates the correct pronunciation via the relevant articulatory instructions and this, in turn, can be fed back to the phonological system via the spoken input system to establish if the two characters share a representation. The reason why any confusion between 猫 and 毛 is maintained even when the characters are spoken aloud is that the matching of their pronunciations is performed on the basis of the information in the phonological system, which is where the two characters share the same unit. That is, the homophony judgment is based on the same abstract phonological information in both the silent and aloud tasks.

In order to perform the single-character homophone judgment task, it is necessary to establish whether there are any other concepts associated with the phonological representation of the presented character. If the phonological representation includes the syllable separated from its tone, then 肉 (i.e., *ròu*) will share that syllable representation with 柔 (i.e., *róu*) and this will make it harder to decide that the former has no homophonic partner compared to 丢 where the atonal syllable is unique.

It is interesting to note that the single-character homophone judgment task is extremely difficult for Mandarin speakers. The *ròu*-type of non-homophones were misclassified as homophones 61.5% of the time (averaged across the silent and aloud conditions) and even the *diū*-type of non-homophones were misclassified 46.4% of the time. In marked contrast, the equivalent task in English is very easy, with Taft (1984) reporting less than 2% misclassifications of non-homophones (e.g., *NEAT*) as homophones. A feasible explanation for the difficulty in Mandarin lies in the fact that the connection between orthography and phonology is arbitrary for logographic characters because there are no sublexical links (Taft and Chen, 1992). When there are sublexical links, such as in English, rebound between the sublexical orthographic and phonological units indicates what any homophones are likely to look like. That is, when *NEAT* is presented, the grapheme unit *EA* will activate the phoneme unit /i:/ which can, in turn, send activation back to other graphemic units linked to it, such as *EE*. Similarly, the grapheme unit *N* will activate the phoneme unit /n/ which can rebound back to the grapheme unit *KN*. Thus, it can be established what the options are for other words pronounced identically with *NEAT* (e.g., *NEET*, *KNEAT*, *KNEET*, etc). When these fail to activate a word-level unit, a non-homophone decision can be made. In Mandarin, however, any character could be a homophone of any other character. It is impossible to narrow down the possibilities and, as a result, it is hard to decide that no other character is linked to the pronunciation of the character that has been presented.

Within the framework of Figure 3, however, it might seem possible that a homophone judgment could be successfully based on whether more than one concept is activated via the spoken input system. For example, when presented with the character 丢 (*diū*), activation of its phonological representation via the concept will allow its pronunciation to be generated which, in turn, can access the spoken input system. This can then activate all the concepts that are associated with that pronunciation and, if there is only one such concept, it implies that the character is a non-homophone. The evidence suggests, though, that such a pathway is not a reliable means of assessing homophony given that performance with *diū*-type non-homophones was close to chance level. Why might that be?

One clear possibility is that even non-homophones can have multiple meanings and that their pronunciation will activate multiple concepts. For example, 丢 can mean “lose,” “throw,” or “put aside.” As such, it is hard to distinguish the situation where several meanings are associated with only one character from the situation where they are associated with more than one character, hence making homophone judgment difficult. Note that the same spelling can have multiple meanings in English as well (e.g., *PORT* meaning “harbor” and “an alcoholic drink”), but judging homophony is not problematic in this case because, unlike Chinese, the sublexical rebound that can occur between orthography and phonology allows the potential alternative spellings of a word to be identified.

## Explaining results with the Stroop task

The conclusion that homophony between two characters is considered acceptable even when their tones are different, is putatively contradicted by another study using quite a different paradigm. When naming the color in which a character was printed, Spinks et al. (2000) found that that a homophone of a competing color generated greater interference when the tone was correct than when it was not (a type of Stroop effect). For example, the character 栏 (meaning “fence”) is exactly homophonic with the character meaning “blue” (蓝), both being *lán*, while the character 览 (meaning “view”) has a different tone, namely, *lǎn*. In the Spinks et al. (2000) study, these characters were printed in red and participants were asked to name that color (i.e., *hóng*), with Stroop interference being seen as a delay in responding relative to a character that did not share any phonology with a color word. Interference from a homophone of the conflicting color was only seen on response times when the character had the same tone as the conflicting color (i.e., 栏, and not 览), suggesting that tone is an integral part of the syllable.

Such a conclusion is unwarranted, however. When 栏 is presented, it will activate its phonological representation via the concept level and its pronunciation will be generated from this (i.e., [lán]), especially since the task necessarily engages the overt pronunciation system in order to name the color in which the character is printed. When this pronunciation is fed into the spoken input system, it will activate all concepts that are pronounced [lán], including the concept “blue.” As such, conflict can occur with the concept “red,” hence leading to a delay in naming the color in which 栏 is printed (i.e., *hóng*, “red”). On the other hand, the overt pronunciation [lǎn] will be generated when 览 is presented, which will not activate a color concept. Therefore, even if the phonological representations for 览 (“view”) and 蓝 (“blue”) have a shared element, namely, the atonal syllable /lan/, this will not lead to a delay in the Stroop task because the relevant conflict occurs at the concept level rather than in the phonological system as in the homophone judgment task. Such a difference between the outcome of the two tasks reinforces the explanatory power of a model like that depicted in Figure 3 by clarifying what the processing steps might be for the particular task.

A further finding of Spinks et al. (2000) is also worth noting. When the presented character was homophonic with the correct print color (e.g., red), responses were facilitated regardless of whether the tone was correct or not (e.g., both 洪, *hóng*, “flood” and 轰, *hōng*, “boom”). It seems that activation of the concept “red” by the homophonic character 洪 through the spoken input system does not add to the ability to name the color red compared to when that concept is not activated (i.e., by the character 轰). Response times are only affected at the concept level when there is a conflict between competing responses, not when they are congruous. Instead, it might be suggested that the facilitation from congruency is a result of the shared articulatory instructions between *hōng* and *hóng* arising at the time of overt pronunciation. Having said this, however, more recent studies using other measures of speech production (e.g., Chen et al., 2002; Chen and Zhang, 2025) have observed greater facilitation of pronunciation when a homophonic prime shares its tone with the target than when it does not, which is inconsistent with the equal facilitation reported by Spinks et al. (2000). How these different findings are to be reconciled remains unclear at this stage.

## The role of pinyin in the phonological representation

If it is indeed the case that the phonological representation of Mandarin separates the tone from the rest of the syllable, we can ask why this might come about. After all, the tone is an integral part of the pronunciation of a morpheme, so why is it not incorporated into the phonological representation of the morpheme? What can be suggested is that it is the exposure to pinyin that alerts Mandarin speakers to the separation of the tone from the segments because they are represented quite differently in pinyin (see Taft, 2024). That is, the segments are represented by letters while the tone is represented by a non-verbal shape (i.e., a line).

One way to examine the role of pinyin in molding the phonological representation of Chinese would be to give the homophone judgment task to Chinese speakers who have not learnt pinyin, for example, Cantonese speakers from Hong Kong especially prior to the widespread use of alphabetically mediated texting (e.g., Chen and Yuen, 1991). Such a study was indeed reported by Taft and Chen (1992) using the two-character matching task described earlier where Mandarin speakers showed a difference between the tone-different and vowel-different conditions of 129 ms for speed and 28.2% for accuracy (averaged across the silent and aloud versions of the task). In the Cantonese version of this task, it was found that the difference between the two conditions was 87 ms for speed but only 0.9% for accuracy. So, while the data indicate that Cantonese speakers do show a delay in deciding that two characters are not homophonic when their tones differ, the contrast with Mandarin speakers is quite dramatic in terms of accuracy.

It can therefore be argued that the nature of Mandarin and Cantonese representation is qualitatively different somewhere in lexical memory, and it is being suggested here that the phonological system is the locus for this. In particular, Mandarin separates the segments from the tone in the phonological system as a result of learning pinyin whereas Cantonese integrates the tone with the vowel (as in Figure 2). Thus, the erroneous matching of two morphemes that differ in tone occurs to a considerable extent in Mandarin but not at all in Cantonese. The significant effect on response times in Cantonese can be explained in terms of a delay arising from the comparison of surface forms, where the articulatory instructions are more similar for two syllables that differ in their tone than for two syllables that differ in their vowel.

There is another series of studies into the processing of Cantonese tone, however, that requires comment. This is the research of Wong and H.-C. Chen using the picture-word interference task (see Wong et al., 2023, for an overview) where a picture was to be named while a distracting word was visually or aurally presented. The relationship between the first syllable of this word with the first syllable of the name of the picture was manipulated, including where these syllables were homophones with or without the same tone (i.e., tonal or atonal homophones). Picture naming responses were facilitated relative to an unrelated distractor condition for both types of homophone, but significantly more so when the tone was the same. Such a result is comparable to what Chen et al. (2002) found in Mandarin using the implicit priming task and, therefore, throws doubt on the idea that the two Chinese languages are processed differently. Against this, though, Chen and Zhang (2025) reported a pattern of neurophysiological data in a Mandarin version of the picture-word interference task that was different to the pattern reported by Wong et al. (2023) in Cantonese and suggested that this discrepancy might have arisen from the widespread use of pinyin in Mandarin instruction in contrast to the unsystematic exposure to an alphabetic script when learning Cantonese. Consistent with what is being proposed here, Chen and Zhang (2025) argue that pinyin represents the tones separately from the rest of the syllable, hence alerting the Mandarin speaker to their existence as a separate entity.

The strongest conclusion from the comparison of Mandarin and Cantonese is that only the former separates the tone from the syllable in the phonological representation. However, the fact that production of Cantonese words is influenced by atonal homophones might suggest that there is at least some separation of the tone from the syllable in Cantonese, even if not to the same extent as in Mandarin. While it is conceivable that the atonal homophone effect in Cantonese (Wong et al., 2023) arose from the shared articulatory instructions between the pronunciation of the picture and the distractor word, a similar argument could be made to explain the atonal homophone effect in Mandarin implicit priming (Chen et al., 2002). Therefore, there is uncertainty in the comparison of Mandarin and Cantonese as a means of determining whether pinyin knowledge influences the representation of tone. Indeed, there might be other reasons for Cantonese to integrate tone and segmental information more than Mandarin such as the fact that it has a more complex tonal system.

As such, other approaches to determining the influence of pinyin might prove more fruitful. For example, the finding of Yin et al. (2011) that knowledge of pinyin amongst 8-to 9-year-old Mandarin speakers is correlated with tone awareness implies that pinyin does alert the Mandarin speaker to the tone as an entity separate to the syllable. In addition, a study of dichotic listening by Shao et al. (2022) observed a difference in tone processing between Cantonese speakers who had learnt an alphabetic version of Cantonese that represented tonal information (i.e., “Jyutping”) and those who had not, a result that also indicates that learning a mediating alphabetic script can modify the way in which tones are integrated with segmental information.

## Non-native Mandarin learners

If it is true that tones and segments are stored separately in the phonological system of native Mandarin speakers as a result of exposure to pinyin, then this will certainly be true for non-native speakers as well. It is almost always the case that pinyin is used as the medium for the formal teaching of Mandarin. The difference between the native and non-native speaker lies in the former being able to readily link the tone with the rest of the syllable, unlike the latter. In particular, while the tone and the rest of the syllable are represented separately in the phonological system for both native and non-native speakers, the spoken input system of the native speaker has a representation of the integrated pronunciation which is linked to the phonological system, both directly and via the concept level, in such a way that it ties the tone to its appropriate segmental information. This can be seen in Figure 3 where the spoken input representation [māʊ] has links to the relevant representations in the phonological system including the tone, hence binding them together. Such links also exist from the concept to the phonological system. In contrast, it is very hard for someone learning Mandarin to process the tone of a spoken morpheme, especially when they are from a non-tonal language background (e.g., Kiriloff, 1969), and this will lead to considerable difficulty in setting up a spoken word representation that includes the tone.

As a result, there are three areas in which a Chinese language learner is likely to have difficulty with regard to tones. First, as just mentioned, those from a non-tonal language background simply fail to identify the tone of a spoken word because, in their first language, tonal variation has no impact on the meaning of the word and is therefore largely ignored. So, this is a problem with the spoken input system, and several proposals have been explored for overcoming it (e.g., Godfroid et al., 2017; Liu et al., 2011). The second difficulty lies in the ability to produce the correct tone when speaking Mandarin and attempts have been made to teach mastery of tonal pronunciation (e.g., Třísková, 2017) by seeking to improve the accuracy of carrying out articulatory instructions. However, even if the learner were able to pronounce tones like a native speaker, there is a third, more critical problem that they have to overcome in order to communicate effectively. This is the knowledge of which tone is the correct one for any particular morpheme.

When learning a new word, it is necessary to associate the correct tone with the rest of the syllable. The problem for the non-native learner is that the links connecting the spoken input with the phonological system must bring together the segmental information with the appropriate tone (as seen in Figure 3), and this is difficult to achieve because the tone is hard to represent in the spoken input for someone with a non-tonal background. Instead, the main basis for knowing what the correct tone is for a particular morpheme is to remember which diacritic mark was used in the pinyin version of that morpheme, and it is hard to remember the pairing of two items when each is represented in a different form (i.e., a grouping of letters and a non-verbal line). Even if the learner has learnt to differentiate tones in the speech signal, there is still the problem of binding the correct tone and segmental information together for any particular syllable.

Taft (2024) explicitly addresses this problem by developing an alphabetic script for learning Mandarin that incorporates the tone into the syllable in the form of another letter following the vowel (e.g., an *h* is added after the vowel for Tone 2 giving *rouh* instead of *róu*, and *y* is added after the vowel for Tone 4 giving *rouy* instead of *ròu*). Taft (2024) showed that learners were able to produce the correct tone (and indeed the complete syllable) more often when taught using this tonal spelling method than when taught standard pinyin.^5^ In other words, the tonal spelling method replaced the separate representations for segmental information and tone in the phonological system with a single representation that integrated the two by coding them in the same way (i.e., as letters). It is even possible that being able to link the tone to its relevant segments in the phonological system, itself leads to a representation in the spoken input system that captures the appropriate tone, as is the case for native speakers, and this is something that is yet to be established.

## Conclusion

The purpose of this paper is to present a general framework for thinking about the way in which Chinese is represented in lexical memory with a particular focus on visual word processing. A more comprehensive model using this framework would include details about the role of radicals in the orthographic processing of characters (see Taft, 2006b) as well as the way in which characters are combined to create compound words (e.g., Wei et al., 2023). The aim here, however, is to provide a concrete account of the various pathways that are available to the reader and also the general nature of the representations that are drawn upon.

It is argued that acquisition of pinyin when learning to read, changes the way words are represented in the phonological system and leads to the separation of the tone from segmental information. In addition, the concept level mediates between orthography and phonology, with the latter being seen as an output system generating spoken words via articulatory instructions. Speech is recognized through a separate spoken input system which directly activates the concept level.

A fundamental qualitative difference between a Mandarin learner and a native speaker, especially when the former has a non-tonal first language, is the existence of a spoken input system that is sensitive to tone. As a result of their early and sustained exposure to spoken Mandarin, the native speaker is able to set up a representation in the spoken input system that integrates the tone with the rest of the syllable. The non-native learner has difficulty in doing that and, instead, develops their vocabulary through the use of pinyin whereby it is hard to develop a representation that connects segmental information with the appropriate tone. A way of circumventing this problem through tonal spelling is proposed which allows the development of a phonological representation that integrates the tone and the segments. Whether this will eventually allow the non-native speaker to attain a lexical processing system that emulates the native system (as depicted in Figure 3) remains to be seen.

## Data Availability

The original contributions presented in the study are included in the article/supplementary material, further inquiries can be directed to the corresponding author.

## Footnotes

1 Note that the term “concept” is referred to in other versions of this model as a “lemma” (e.g., Taft, 2006b), which is not a semantic unit *per se*, but rather, an abstract unit that mediates between form (i.e., orthography and/or phonology) and meaning.

2 See Taft and Zhu (1995) for evidence for this rather than there being a whole-word representation in the orthographic lexicon.

3 The possibility that this is also the case for alphabetic scripts is beyond the scope of the present paper and will not be pursued here (but see Taft and van Graan, 1998).

4 No commitment is made to how the tone might be represented in the phonological system. Nevertheless, it seems more parsimonious to suggest that the differentiation between tones is the same across all four tones and that, if the greater physical similarity between one tone and another has an impact on processing, this only happens once the syllables are articulated. The adjustment of Tone 3 to Tone 2 before another Tone 3 syllable (i.e., tone sandhi), would also happen at the late output stage.

5 The tonal representation proposed by Taft (2024) was not compared to pinyin when using numbers instead of diacritics (e.g., mao 1). However, given that numbers are of a different category to letters and, hence, no more easily integrated than are diacritics, he suggests that there is no reason for the advantage observed for the alphabetic tonal representation to be any different, but this is something that could be tested empirically.
